# Peptide transporter2 (PTR2) enhances water uptake during early seed germination in *Arabidopsis thaliana*

**DOI:** 10.1007/s11103-020-00967-3

**Published:** 2020-01-29

**Authors:** Myoung-Goo Choi, Eui Joong Kim, Ji-Young Song, Sang-Bong Choi, Seong-Woo Cho, Chul Soo Park, Chon-Sik Kang, Youn-Il Park

**Affiliations:** 1 Department of Biological Sciences, Chungnam National University, Daejeon, 34134 Republic of Korea; 2 National Institute of Crop Science, Rural Development Administration, Wanju, 55365 Republic of Korea; 3 Division of Bioscience and Bioinformatics, Myongji University, Yongin, 17058 Gyunggi-do Republic of Korea; 4 Department of Crop Science and Biotechnology, Chonbuk National University, Jeonju, 54896 Republic of Korea

**Keywords:** ABA, ABI4, Arabidopsis, Peptide transporter 2, Seed germination, Water uptake

## Abstract

**Key message:**

PTR2 in *Arabidopsis thaliana* is negatively regulated by ABI4 and plays a key role in water uptake by seeds, ensuring that imbibed seeds proceed to germination.

**Abstract:**

Peptide transporters (PTRs) transport nitrogen-containing substrates in a proton-dependent manner. Among the six PTRs in *Arabidopsis thaliana*, the physiological role of the tonoplast-localized, seed embryo abundant PTR2 is unknown. In the present study, a molecular physiological analysis of PTR2 was conducted using *ptr2* mutants and *PTR2CO* complementation lines. Compared with the wild type, the *ptr2* mutant showed ca. 6 h delay in testa rupture and consequently endosperm rupture because of 17% lower water content and 10% higher free abscisic acid (ABA) content. Constitutive overexpression of the *PTR2* gene under the control of the Cauliflower mosaic virus (CaMV) *35S* promoter in *ptr2* mutants rescued the mutant phenotypes. After cold stratification, a transient increase in *ABA INSENSITIVE4* (*ABI4*) transcript levels during induction of testa rupture was followed by a similar increase in *PTR2* transcript levels, which peaked prior to endosperm rupture. The *PTR2* promoter region containing multiple CCAC motifs was recognized by ABI4 in electrophoretic mobility shift assays, and *PTR2* expression was repressed by 67% in *ABI4* overexpression lines compared with the wild type, suggesting that PTR2 is an immediate downstream target of ABI4. Taken together, the results suggest that ABI4-dependent temporal regulation of *PTR2* expression may influence water status during seed germination to promote the post-germinative growth of imbibed seeds.

**Electronic supplementary material:**

The online version of this article (10.1007/s11103-020-00967-3) contains supplementary material, which is available to authorized users.

## Introduction

Seeds comprise the seed coat, endosperm, and embryo. Germination of dormant seeds commences with the uptake of water and terminates with the emergence of the radicle through the seed coat (Bewley 1997). During germination, increase in the total water content of seeds follows a classic triphasic model, where the initial and final rapid water uptake periods (I and III, respectively) are intervened by the slow water uptake period II. Water imbibition-induced expansion of the embryo and endosperm leads to testa rupture (TR), which is followed by the protrusion of radicle through the endosperm, which leads to endosperm rupture (ER) (Weitbrecht et al. 2011).

Successful germination and post-germinative growth are partly determined by the endogenous plant hormones, including abscisic acid (ABA), gibberellic acid (GA), ethylene, and auxin, and the antagonism between GAs (which stimulate seed germination) and ABA (which establishes and maintains seed dormancy) is the primary factor that regulates seed germination (Pritchard et al. 2002; Seo et al. 2006). The endogenous level of ABA in dormant seeds declines upon imbibition during cold stratification (Chiwocha et al. 2005). Exogenously supplied ABA inhibits seed germination by affecting ER (Muller et al. 2006). ABA-mediated regulation of seed germination involves ABA receptor complexes consisting of PYRABACTIN RESISTANCE (PYR)/PYR1-LIKE (PYL)/REGULATORY COMPONENT OF ABA RECEPTOR (RCAR), type 2C protein phosphatases (PP2Cs), and SnRK2s (Raghavendra et al. 2010; Weiner et al. 2010). Multiple seed-abundant ABA-responsive transcription factors, including the B3 domain-containing ABA INSENSITIVE3 (ABI3), the APETALA2 (AP2) domain-containing ABI4, and the basic leucine zipper (bZIP) domain-containing ABI5 (Giraudat et al. 1992; Finkelstein et al. 1998; Finkelstein and Lynch 2000), operate in the ABA signaling pathways either in concert or independently. For instance, ABI4 inhibits ABA catabolism via the transcriptional repression of *CYP707A* (Shu et al. 2013) and catabolism of embryonic lipids (Penfield et al. 2006) during the germination process. MYB96 directly regulates *ABI4* expression during embryonic lipid mobilization (Lee et al. 2015).

ABA is catabolized either by 8′-hydroxylation or glycosylation at the carboxyl group. Hydroxylation at the C-8′ of ABA is catalyzed by cytochrome P450-type mono-oxygenases (CYP707As) (Kushiro et al. 2004), and unstable 8'-hydroxy-ABA is then isomerized spontaneously to phaseic acid (Kepka et al. 2011). Glycosylation is catalyzed by eight ABA glycosyltransferases (GTs) in *Arabidopsis thaliana* (Lim et al. 2005). AtBG1 and AtBG2 inactivate ABA by converting it to ABA-glucose ester (ABA-GE) that accumulates in the vacuole or apoplast (Hartung et al. 2002; Lee et al. 2006). In Arabidopsis seeds, ABA metabolism and signaling-related genes are expressed in both endosperm and embryo, although the expression of *ABI4* is higher in the embryo than in the endosperm (Penfield et al. 2006). Additionally, exogenous glucose (Glc) triggers ABA accumulation by activating the expression of *ABA1* (encoding zeaxanthin epoxidase), *ABA2* (encoding xanthoxin dehydrogenase), and *ABA3* (encoding molybdenum cofactor sulfurase) genes, which in turn suppresses germination (Cheng et al. 2002; Price et al. 2003; Bossi et al. 2009).

Seed storage proteins are stored in the protein storage vacuole (or protein bodies), which is formed from vacuoles during seed maturation as a result of protein deposition and water displacement (Otegui et al. 2006). During seed imbibition and germination, storage proteins are hydrolyzed, and vacuoles fuse with each other, forming a central, lytic vacuole (Hunter et al. 2007; Zheng and Staehelin 2011). Once proteins are hydrolyzed, free amino acids and oligopeptides are transported to the cytosol by peptide transporters (PTRs), a type of symporter proteins, that cotransport protons (H^+^) and a wide range of nitrogen (N)-containing substrates, including nitrate, amino acids, and di-and tri-peptides (Chiang et al. 2004; Tsay et al. 2007), as well as GA, ABA, and jasmonates (Chiba et al. 2015). Among the six di- and tri-peptide transporting PTRs in Arabidopsis, PTR1 and PTR5 localize at the plasma membrane and perform distinct physiological functions; PTR1 regulates N uptake by the root, whereas PTR5 facilitates peptide transport to the germinating pollen (Komarova et al. 2008). *PTR2* is highly expressed in the embryo (Rentsch et al. 1995; Song et al. 1996; Chiang et al. 2004; Léran et al. 2015) and endosperm (Dekkers et al. 2013), and localizes at the tonoplast (Komarova et al. 2012). Antisense suppression of *PTR2* affects flowering and seed development but hardly affects seed germination (Song et al. 1997).

In the present study, we investigated the physiological function of PTR2 during early seed germination. The presence of multiple ABI4-binding motifs in the *PTR2* promoter region led us to investigate the role of ABI4 in the regulation of *PTR2* expression. The abundance of *PTR2* transcripts in the endosperm (Dekkers et al. 2013) and embryo (Rentsch et al. 1995) during the early stage of seed germination (Supplementary Fig. S1), and localization of PTR2 at the tonoplast (Komarova et al. 2012) point to a role PTR2 in the regulation of the hydraulic status of germinating seeds. Indeed, the water content was lower in *ptr2* mutant seeds and ABI4 negatively regulated *PTR2* transcription during seed germination.

## Materials and methods

### Plant materials and growth conditions

*Arabidopsis thaliana* ecotype Columbia (Col-0; WT), seven *ptr* mutants (*ptr1–6*) including *ptr2-1* and *ptr2-2* alleles, two *PTR2* complementation lines (*PTR2CO2* and *PTR2CO5*), *abi4* mutant, and *ABI4* overexpressor (*ABI4OE*) were used in this study. Seeds from seed batches grown and harvested at the same time and stored for less than 2 weeks were treated with 70% ethanol for 1 min and then sterilized with 0.8% sodium hypochlorite. After washing five times with sterilized distilled water (SDW), seeds were imbibed in water in the dark at 4 °C for 2 days to break seed dormancy and then transferred to continuous light (30 µmol m^−2^ s^−1^) at 22 °C. Seeds were sown in SDW supplemented with 2% Glc, 1 µM ABA, 50 µM GA, ABA inhibitors (20 µM nordihydroguaiaretic and 10 µM diniconazole), 20 mM glycine di-peptide (Gly-Gly), 20 mM leucine di-peptide (Leu-Leu), or 10 ∝g ml^−1^ of a transcription inhibitor, Cordycepin. The osmotic potential of SDW was adjusted to − 0.23, − 0.39, − 0.68, or − 0.91 Mpa with polyethylene glycol 4000 (PEG4000; Sigma-Aldrich).

### Characterization of ***ptr*** mutants

T-DNA insertion mutant lines of all six Arabidopsis *PTR* genes were identified at The Arabidopsis Information Resource (TAIR) database (https://www.arabidopsis.org/index.jsp). Seeds of *ptr1-1* (SLAK_131530), *ptr2-1* (SALK_400_D08), *ptr2-2* (SAL_65_B10), *ptr3-2* (SALK_097591), *ptr4-1* (SALK_062626), *ptr5-2* (SALK_116120), and *ptr6-1* (SALK_149283) were obtained from the Arabidopsis Biological Resource Center (ABRC) at Ohio State University, OH, USA. To map the T-DNA insertion sites, genomic DNA was isolated from the leaves of mutant plants using lysis buffer [200 mM Tris–HCl (pH 7.4), 250 mM NaCl, 25 mM EDTA (pH 8.0), and 0.5% sodium dodecyl sulfate (SDS)]. Reverse transcription PCR (RT-PCR) was carried out using the SALK LBa1, LBba1, and LB1 primers and *PTR2* gene-specific primers (Supplementary Table S1). The resulting PCR products were sequenced and compared with the genomic sequence of each gene to map the T-DNA insertion site (Supplementary Fig. S2).

### Generation of ***PTR2CO*** complementation lines

The coding sequence of *PTR2* was amplified by PCR using sequence-specific primers (Supplementary Table S1). The PCR product was digested with *Xba*I and *Bam*HI restriction endonucleases and cloned into the pJJ461 vector. The construct was confirmed by sequencing and transformed into *Agrobacterium tumefaciens* strain GV3101 by the freeze-thaw method. Transformation of *ptr2* mutants was conducted using the floral dip method. *PTR2CO* lines with a single-copy of the hygromycin resistance (*HygR*) gene and *PTR2* were identified by RT-PCR (Supplementary Fig. S2).

### Extraction and analysis of seed storage proteins

Seed proteins was extracted using the chloroform-assisted phenol extraction (CAPE) method. Briefly, seeds (0.1 g fresh weight) were homogenized using a cold pestle and mortar in 1.0 ml buffer [0.25 M Tris–HCl (pH 7.5), 1% SDS, 14 mM dithiothreitol (DTT), and protease inhibitor cocktail (Roche)]. The homogenate was centrifuged at 12,000×*g* for 10 min. Then, 600 µl of the supernatant (embryo extract) was transferred to an Eppendorf tube, followed by the additional of an equal volume (600 µl) of chloroform. The mixture was mixed thoroughly by shaking for 10 min at room temperature. After the addition of 600 µl of buffered phenol (pH 8.0), the organic phase was transferred to two new 2.0 ml Eppendorf tubes (300 µl each). Then, five volumes of methanol containing 0.1 M ammonium acetate were added to each tube, and the mixture was incubated at − 20 °C for 1 h to precipitate the proteins. Proteins were separated by centrifugation at 15,000×*g* for 10 min and washed first with 100% cold acetone and then with 80% cold acetone. The protein pellet was air dried and resuspended in SDS sample buffer [50 mM Tris–HCl (pH 6.8), 100 mM DTT, 2% SDS, 0.1 bromophenol blue, and 10% glycerol]. Proteins were separated by SDS-polyacrylamide gel electrophoresis (PAGE) using 15% resolving and 5% stacking polyacrylamide gels.

### Quantitative real-time PCR (qRT-PCR) analysis

Total RNA was extracted from dry and imbibed seeds using the Spectrum™ Plant Total RNA Kit (SIGMA) and treated with DNaseI (Takara). After NucleoSpin RNA Cleanup (Macherey-Nagel), cDNA was synthesized from 500 ng of total RNA using the iScript cDNA Synthesis Kit (Bio-Rad) and amplified by qRT-PCR on the CFX96 Real Time System (Bio-Rad) using the iQ™ SYBR Green Supermix (Bio-Rad), according to the manufacturer’s instructions. Reactions were performed in triplicate in a 10 µl volume, containing 5 µl SYBR Green Master Mix, 0.5 µl of each primer (10 pmol), 4 µl of 20-fold diluted cDNA, and 0.5 µl nuclease-free water, using the following conditions: 95 °C for 15 min, followed by 40 cycles of amplification at 94 °C for 10 s and 62 °C for 30 s, and lastly a final extension at 72 °C for 30 s. Fluorescence was measured at the end of each extension step. Amplification was followed by melting curve analysis, with continual fluorescence data acquisition during the 65 °C to 95 °C transition. A negative control containing water instead of cDNA template was included in each run. Raw data were analyzed with the CFX Manager software, and gene expression was normalized to the expression of *AP2M* (at5g46630) gene (Czechowski et al. 2005; Dekkers et al. 2012) to minimize variation in cDNA template levels. For each gene, a standard curve was generated using serial dilutions of cDNA, and the resultant PCR efficiency varied from 90 to 99.5%. To ensure that transcripts of single genes were amplified, qPCR amplicons were sequenced. Relative expression levels were calculated using the comparative threshold (Ct) cycle values, based on the 2^−ΔΔCt^ method.

### Quantification of seed ABA and water contents

Imbibed or germinating seeds (20 mg) were frozen in liquid N and ground using a pestle and mortar. The ground powder was immersed in 1 ml of 80% methanol and stored at 4 °C for 1 h. The extracts were filtered with C18 (Sep-Pak Vac) cartridges (Waters, USA) to remove pigments and other polar materials. The filtered solution was dried and concentrated using a rotary vacuum concentrator and suspended in Tris-buffered saline (TBS). The level of ABA was determined using the enzyme-linked immunosorbent assay (ELISA) Kit (Agdia). To measure the seed water content, ca. 330 seeds immersed in SDW were collected at a given time, and external liquid was removed using a silica-based membrane column by spinning at 12,000 rpm for 5 min. Then, dry seed weight was subtracted from the fresh seed weight to determine the amount of water absorbed by the seeds. External osmotic potential of varying concentrations of PEG4000 was calculated from the estimated osmolality (mol kg^−1^) (Money 1989).

### Electrophoretic mobility shift assay (EMSA)

Glutathione S-transferase (GST)-tagged DNA-binding domain of ABI4 (GST-ABI4) was expressed in *Escherichia coli* and purified using a metal-affinity resin (Qiagen) (Finkelstein et al. 2011). Biotin-labeled *PTR2* promoter fragments (− 360 to − 155 bp [P1]; − 760 to − 440 bp [P2]; − 1042 to − 739 bp [P3]; − 1775 to − 1583 bp [P4]) and competitor DNA fragments, including specific unlabeled fragment and *ABI5* promoter fragment (− 915 to − 739 bp; positive control), were used with the purified GST-ABI4 protein in EMSAs (Chemiluminescent Light Shift EMSA kit; Pierce Biotechnology).

### Measurement of GUS activity and transient gene expression

*PTR2* and CaMV *35S* promoters were cloned and fused to the *β-glucuronidase* (*GUS*) reporter gene in pCAMBIA3301 using primers listed in Supplementary Table S1. The resulting constructs were transiently transformed into the WT, *abi4*, and *ABI4OE* lines. Four-day-old seedlings grown in darkness were vacuum-infiltrated with *Agrobacterium* twice for 1 min each (Marion et al. 2008). The samples were incubated in the dark for 1 day and then grown under white light for 2 days. GUS activities were measured using a spectrofluorometer (LS-55; Perkin-Elmer) with the substrate 4-methylumbelliferyl-β-d-glucuronide (Sigma-Aldrich); 4-methylubelliferone (Sigma-Aldrich) was used for calibration. Protein content was determined using bovine serum albumin as the standard (Jaquinod et al. 2007). To perform GUS histochemical staining, germinating seeds were fixed by immersing in 90% (v/v) acetone. The seeds were then washed twice with a solution containing 50 mM sodium phosphate (pH 7), 0.5 mM K_3_Fe(CN)_6_, and 0.5 mM K_4_Fe(CN)_6_, and subsequently incubated in a staining solution containing 1 mM 5-bromo-4-chloro-3-indolyl-β-d-glucuronide (Duchefa) (Lee et al. 2015).

### Statistical analysis

Data (mean ± SE; *n* = 3–5) were analyzed using the two-tailed Student’s *t *test, after normality assessment. Means with *p *values less than 0.05 were considered statistically significant.

## Results

### Abundance of ***PTR2*** transcripts, amino acids, and storage proteins in Arabidopsis seeds during germination

**Fig. 1:**
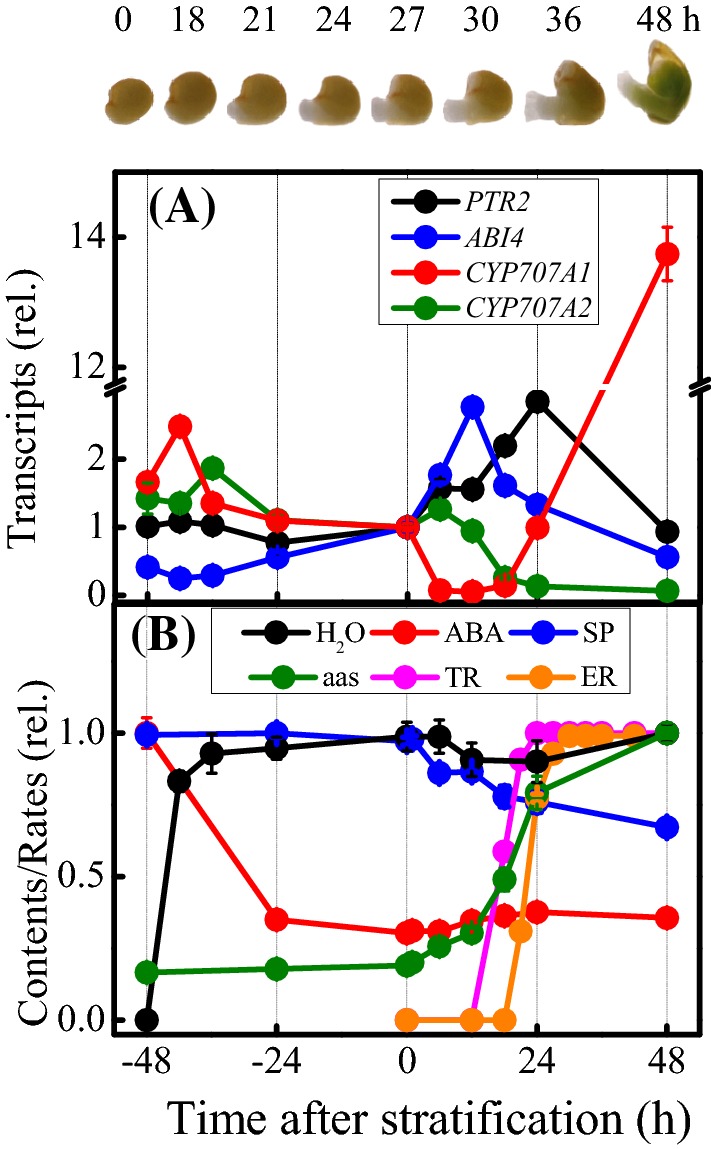
Seed germination kinetics in Arabidopsis. **a** Abundance of ABI4, PTR2, and CYP707A1/A2 transcripts in germinating Arabidopsis seeds. Transcript levels of each gene in germinating seeds were normalized to transcript levels in seeds immediately after stratification. **b** Changes in water, ABA, soluble protein (SP), and amino acid (aa) contents and rates of testa rupture (TR) and endosperm rupture (ER). Water, ABA, SP, and aa contents at a given time were normalized to those of dry seeds (ABA and SP) or post-germinated seeds (water and aa), respectively. ABA, 2.69 ± 0.14 pmol mg^−1^ seed DW; SP, 0.99 ± 0.01 mg mg^−1^ seed DW; aa, 0.68 ± 0.07 µmol mg^−1^ seed DW. The proportion of seeds with testa rupture (TR) or endosperm rupture (ER) was calculated using approximately 100 seeds at a given time. Images of germinating seeds at different stages, including no rupture, testa rupture (TR) with endosperm layer exposed, and endosperm rupture (ER) with radicle protruded, at various time points are provided on the top. Data represent mean ± standard error (SE; n = 3–4). Significant differences were detected at p < 0.05

Publicly available microarray data show that transcript levels of *PTR2* are highly abundant in both dry and germinating seeds, whereas those of the remaining five *PTR* genes are negligible (Supplementary Fig. S1A; Rentsch et al. 1995; Narsai et al. 2011; Dekkers et al. 2013, 2016). In this study, the transcript level of *PTR2* in dry seeds was high and remained almost unchanged during cold stratification at 4 °C for 2 days but showed a transient increase during germination (Fig. 1a). This transient increase in *PTR2* expression was inhibited by the transcription inhibitor cordycepin (Supplementary Fig. S1B). Thus, PTR2 activity appears to be regulated at the post-transcriptional level during early seed germination and then at the transcriptional level during late seed germination.

The tonoplast-localized PTR2 would transport di- and tri-peptides, stored in protein bodies during seed development, to the cytosol. PTR2 would also transport small peptides generated from the degradation of seed storage proteins in embryo or cotyledons (Guerche et al. 1990; Pang et al. 1988) by proteases regulated at the post-transcriptional and/or post-translational level. If this is the case, a substantial amount of free amino acids and small peptides would be expected in dry and imbibed seeds, and a reverse change in free amino acid and storage protein contents would be expected when storage proteins undergo mobilization to support seed germination and subsequently seedling growth. The major seed storage proteins of Arabidopsis include the *α*- and *β*-subunits of the legumin-type 12S globulins (Pang et al. 1988) and the L- and S- subunits of 2S albumin (Guerche et al. 1990) (Fig. 1b). We observed that the contents of globulins and albumins stored in seeds steadily decreased, whereas those of free amino acids increased shortly after stratification but ahead of TR, as expected. However, the inverse change between proteins and amino acids was not observed; instead, the protein and amino acid contents remained unchanged during stratification.

We also quantified the water and ABA contents of germinating seeds, as aquaporin-independent uptake of water (Van der Willigen et al. 2006; Obroucheva 2012) and reduction of ABA content (Chiwocha et al. 2005; Muller et al. 2006) are major events during stratification. During water uptake phase II under low temperature, the ABA content of seeds decreased to the lowest level 1 day prior to the start of the decline in seed storage protein content, and then remained almost unchanged until ER. This decrease in the ABA level is mostly due to the transcriptional activation of *CYP707A1* and *CYP707A2* (Fig. 1a; Dekkers et al. 2013). These changes in *CYP707A1* and *CYP707A2* transcript levels after stratification were cordycepin-sensitive (Supplementary Fig. S1B), suggesting a transcriptional regulation of both these genes during seed germination. However, transcripts of *CYP707A3*, *CYP707A4*, *GT*s, and *BG*s showed minimal or no changes (data not shown).

### Mutation of ***PTR2*** delays TR

**Fig. 2:**
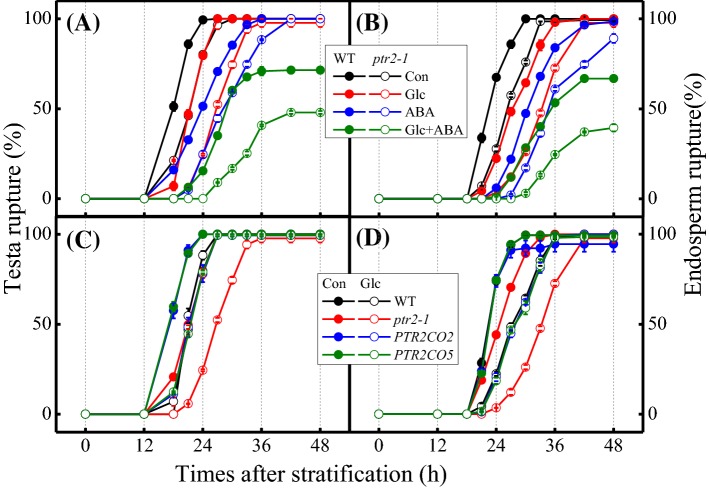
Delay in testa rupture (TR) and endosperm rupture (ER) in *ptr2* mutant seeds. **a**, **b** Additive effects of ABA and glucose (Glc) on the germination of *ptr2* mutant seeds. Col-0 (wild type; WT) and *ptr2-1* mutant seeds were germinated on sterile distilled water (SDW; Con), 2% Glc, 1 µM ABA, or 2% Glc + 1 µM ABA. **c**, **d** Complementation of the germination delay phenotype of *ptr2-1* mutant seeds. Seeds of Col-0 (WT), *ptr2-1*, and *PTR2CO2* and *PTR2CO5* complementation lines were germinated on SDW. Data represent mean ± SE (*n* = 3–4)

To identify the physiological role of PTR2 in the seed germination process, *ptr2* mutants were treated with Glc, ABA, and GA either alone or in combination. The germination of both WT seeds started at 12 h after stratification (HAS) and was almost complete at 36 HAS (Figs. 1, 2). However, *ptr2* mutant seeds displayed an approximately 6 h delay mainly because of delayed TR (Fig. 2a), and consequently delayed ER, albeit to a lower extent (Fig. 2b). This delayed TR phenotype of the *ptr2* mutant could not be recovered by the exogenous application of GA (Supplementary Fig. S3A) or amplified by that of ABA and Glc (Fig. 2a, b). Additionally, the delay in TR was similar between the allelic mutants, *ptr2-1* and *ptr2-2* (Supplementary Fig. S3B), and was fully rescued by the overexpression of *PTR2* under the control of the CaMV *35S* promoter (*35SCaMVp::PTR2*) in the complementation lines *PTR2CO2* and *PTR2CO5* (Fig. 2c, d). Furthermore, absence of TR delay in other *ptr* mutants strongly suggests a unique role of PTR2 in seed germination (Supplementary Fig. S3B).

### Reduction in the water content of ***ptr2*** mutant seeds during germination

**Fig. 3:**
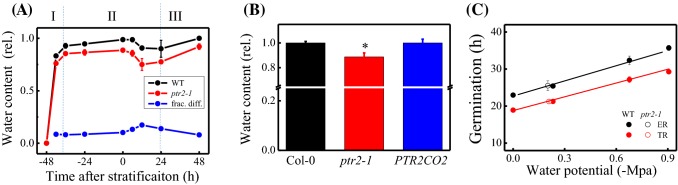
Role of PTR2 in water uptake during seed germination. **a** Changes in water content were analyzed during the progression of seed germination. Col-0 (WT) and *ptr2-1* mutant seeds were germinated on SDW (Con). Fractional difference is the difference in water content at a given time point between WT and *ptr2-1* mutant seeds. Data represent mean ± SE (*n* = 3–4).**b** Complementation of the water deficit phenotype of the *ptr2* mutant. Seeds of Col-0 (WT), *ptr2-1* mutant, and*PTR2CO2* complementation line were used 12 h after stratification. Data represent mean ± SE (*n* = 3–4). Statistically significant differences are indicated with an asterisk (*p < 0.05).**c** Estimation of the water potential of the *ptr2* mutant during seed germination. Water potential was calculated based on the linear positive correlation between the water potential generated by PEG4000 and time needed to reach 50% TR (r^2^ = 0.992) or ER (r^2^ = 0.995). Data represent mean ± SE (*n* = 3–4)

In Arabidopsis, swelling of the embryo and endosperm due to imbibition leads to the mobilization of storage reserves and translation of de novo synthesized or stored mRNAs (Weitbrecht et al. 2011), among other complex multiple events, ultimately leading to TR. Therefore, we determined the water content of germinating *ptr2-1* seeds exhibiting the delayed TR phenotype. Rapid water uptake during phase I was not affected by the *PTR2* mutation. However, water uptake during phase II and phase III was lowered by 17% and 8%, respectively (Fig. 3a). This phenotype was rescued by the expression of *35SCaMVp::PTR2* in *ptr2-1* mutant (Fig. 3b), confirming that PTR2 is at least in part involved in water uptake during phases II and III. Osmotic potential contributed by PTR2 was estimated at − 0.21 MPa, based on the time required to reach 50% TR or ER (Fig. 3c).

### Increase in ABA content in ***ptr2*** mutant seeds during the early phase of germination

A linear interaction between ABA contents and water potential during seed germination (Welbaum et al. 1990) implies that *ptr2* mutant seeds with lowered water content should contain more ABA than WT seeds at the same germination stage. Indeed, the ABA content of germinating *ptr2* seeds was 10–16% higher than that of WT seeds as well as those of *PTR2CO2* and *PTR2CO5*. Furthermore, PEG4000 treated WT, *ptr2-1*, and *PTR2CO* seeds showed ca. 60% higher ABA contents than untreated seeds (Table 1). Increase in the ABA content of WT seeds caused by *PTR2* mutation and PEG4000 treatment was comparable to that caused by Glc and diniconazole treatments, respectively. Thus, the *PTR2* mutation seems to impair ABA catabolism during seed germination to a similar extent as that caused by exogenous Glc application.

**Table 1:** Seed ABA contents of Col-0 (wild-type; WT), *ptr2* and *abi4* mutants, PTR2 complementation lines (*PTR2CO2* and *PTR2CO5*), and *ABI4* overexpression line (ABI4OE)

Genotype	Treatment^a^	ABA content (pmol/mg DW)^b^
WT	Con	0.98 ± 0.01
	Dini	1.44 ± 0.02*
	Glc	1.14 ± 0.02*
	PEG4000	1.56 ± 0.04*
*ptr2-1*	Con	1.08 ± 0.04^†^
	Glc	1.36 ± 0.06*
	PEG4000	1.56 ± 0.01*
*PTR2CO2*	Con	0.98 ± 0.02
	Glc	1.36 ± 0.01*
	PEG4000	1.52 ± 0.09*
*PTR2CO5*	Con	0.94 ± 0.01
	Glc	1.04 ± 0.08*
	PEG4000	1.55 ± 0.05*
*abi4*	Glc	0.91 ± 0.02^†^
*ABI4OE*	Glc	1.43 ± 0.01^†^

^a^Seeds were treated with 2% glucose (Glc), 10 mM diniconazole (Dini), or 18.9% polyethylene glycol (PEG) 4000, equivalent to − 0.68 Mpa, at the beginning of imbibition. *Con* control

^b^Data represent mean ± standard error (SE; *n* = 3–4). Statistical significance of the differences between control and Glc/PEG4000 treated seeds of a genotype is indicated with an asterisk (*p < 0.05), and statistical significance of the differences between WT and mutant/CO/OE seeds is indicated with ^†^(*p* < 0.05). *DW* dry weight

### ABI4 acts as a repressor of ***PTR2***

**Fig. 4:**
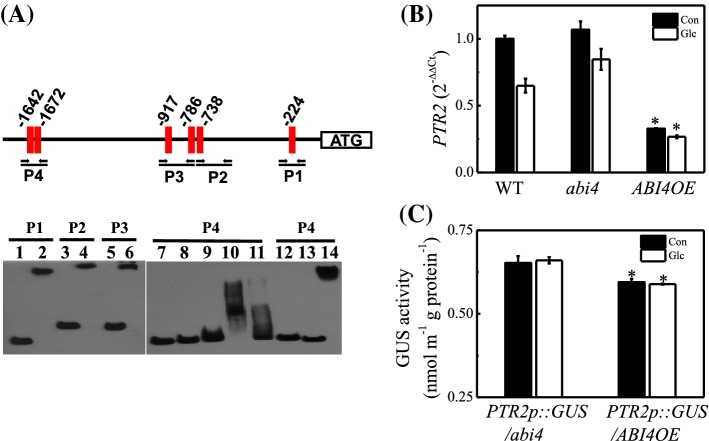
Repression of *PTR2* transcription by ABI4. **a** Schematic representation of the PTR2 promoter containing six CCAC motifs. Four promoter fragments (P1, − 360 to − 155 bp; P2, − 760 to − 440 bp; P3, − 1042 to − 739 bp; P4, − 1775 to − 1583 bp), each containing a single CCAC motif (red square), were used for the electrophoretic mobility shift assay (EMSA). Arrows indicate primers used for cloning. PTR2 promoter fragments (P1–P4) were co-incubated with purified GST-ABI4 (lanes 1–11) and GST-ABI5 (lanes 13 and 14) DNA-binding domain protein, and GST (lane 12). Biotin-labeled DNA fragments (30 fmol) were incubated for 30 min at 22 °C with (lanes 2, 4, 6, 8, 9, 10, 11, 12, and 14) or without (lanes 1, 3, 5, 7, and 13) purified GST-ABI4 (6 μg; lanes 1 to 6, 2 μg; lane 8, 4 μg; lane 9, 8 μg; lanes 10 and 11), GST-ABI5 (6 μg; lane 14; control) or GST (3 μg; lane 12; control). A 200-fold molar excess of the unlabeled P4 inhibitor was included (lane 11). **b** Quantitative real-time PCR (qRT-PCR) analysis of PTR2 expression in Col-0, abi4, and ABI4OE seeds at 12 h after stratification. Seeds were germinated on SDW (Con) or 2% Glc. Data represent mean ± SE (n = 3–4). Statistically significant differences are indicated with an asterisk (*p < 0.05). **c** Transient expression of PTR2 promoter-driven GUS gene (PTR2p::GUS) in abi4 and ABI4OE. GUS activity (nmol min^−1^ μg protein^−1^) was measured using the substrate 4-methylumbelliferyl-β-d-glucuronide. Data represent mean ± SE (n = 3–4). Statistically significant differences are indicated with an asterisk (*p < 0.05)

The *PTR2* promoter contains multiple ABI4-binding motifs of the sequence CCAC (Bossi et al. 2009; Reeves et al. 2011; Fig. 4a). To test whether these CCAC sequences are recognized by ABI4, we conducted EMSAs using four CCAC motif-containing *PTR2* promoter fragments (P1–4) and the GST-ABI4 fusion protein. The recombinant ABI4 protein containing only the DNA-binding domain bound directly to all *PTR2* promoter fragments, like ABI5 (a positive control), but this binding was inhibited by unlabeled, cold fragments (Fig. 4a). If ABI4 acts immediately upstream of *PTR2*, then the transcript level of *PTR2* should be affected in *abi4* as well as in *ABI4OE* plants but in an opposite manner. Compared with the WT, *ABI4OE* plants showed a significantly lower transcript level of *PTR2*, but the *abi4* mutant failed to show a significant enhancement in *PTR2* transcript level (Fig. 4b). This inhibitory effect was also conserved in seeds treated with Glc (Fig. 4b), where endogenous ABA level was enhanced (Table 1). Taken together, these data strongly suggest that *PTR2* transcription is negatively regulated by ABI4. To confirm these results in vivo, *PTR2* promoter was fused to the *GUS* reporter gene (*PTR2p::GUS*) and transformed into hypocotyls of WT, *abi4*, and *ABI4OE*. The expression of *PTR2-GUS* was lowered by 10% in the *ABI4OE* background, consistent with the results of the EMSA, thus confirming that ABI4 represses *PTR2* transcription by directly binding to the CCAC motifs in *PTR2* promoter (Fig. 4c).

## Discussion

**Fig. 5:**
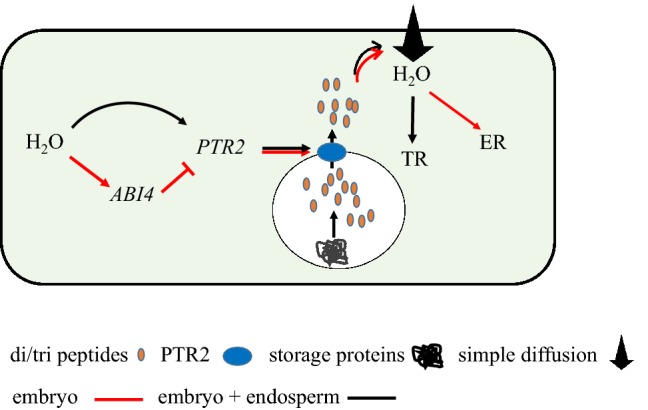
Schematic representation of the role of PTR2 in water uptake during early seed germination in Arabidopsis. In endosperm and embryo tissues, *PTR2* is activated at the post-transcriptional level, whilst its transcriptional regulation confines mostly in the embryo due to the presence of its negative regulator ABI4. Once activated and localized to the vacuolar membrane, PTR2 transports small peptides stored during seed maturation or generated by the enzymatic hydrolysis of seed storage proteins to the cytosol. Water potential generated by the accumulation of small peptides in the cytosol facilitates water uptake by diffusion, which leads to TR and ER in a sequential manner. Arrow and ⊥ indicate activation and repression, respectively

Unlike endospermless species, Arabidopsis exhibits two consecutive germination processes, including TR and ER (Muller et al. 2006). The PTR2 protein is abundant in the embryo (Rentsch et al. 1995; Supplementary Fig. S4) and endosperm (Dekkers et al. 2013). Although mutation of the *PTR2* gene did not affect the canonical triphasic water uptake kinetics, it resulted in lower seed water content, especially during phase II and early phase III. The dependence of TR, and hence ER, on external water potential (Fig. 3c) implies the importance of the endogenous osmotic potential in determining the expansion of the embryo and endosperm tissues. Currently, it is unknown how the biochemical loss of PTR2 is involved in the water-driven seed swelling process. Similar to amino acids, which act as endogenous osmotic solutes like sugars and potassium ions (Bove et al. 2001), di- and tri-peptides seem to also act as endogenous osmotica. Then, increases in the cytosolic level of small peptides via transcriptionally or post-transcriptionally activated PTR2 would increase the cytosolic osmotic potential (to approximately − 0.21 Mpa, as a proxy measure), contributing to aquaporin-independent water diffusion into cells (Fig. 5). Expression of *PTR2p::GUS* in the germinating embryo during phase II (Supplementary Fig. S4), which is shaped by ABI4, suggests that PTR2 is involved in water uptake rather than in N mobilization from the endosperm to the growing axis. Our results are consistent with those of Song et al. (1997); the authors showed that *ptr2* mutant alleles exhibited comparable seedling growth as the antisense mutants.

Di- and tri-peptide sources available for the tonoplast-localized PTR2 seem different between the first half and second half of the water uptake phase II. Time-course analysis of water and storage protein contents of seeds clearly revealed that the inhibition of water uptake observed in the *ptr2* mutant seeds during stratification occurred earlier than the decline in the storage protein content. Thus, PTR2 in seeds seems to initially transport di- and tri-peptides stored in protein storage vacuole or protein bodies to the cytosol, followed by the peptides hydrolyzed from storage proteins. Accordingly, amino acids and small peptides that accumulate in the cytosol would serve primarily as osmotica, contributing to the swelling of the embryo and endosperm, a prerequisite for TR. At the later stage of seed germination and subsequent seedling growth, these amino acids and small peptides would be utilized for further N metabolism. The content and composition of seed storage proteins were comparable among the dry seeds of the WT, *ptr2-1* mutant, and *PTR2OE*s matured at both 22 °C and 16 °C (Supplementary Fig. S5), thus ruling out the role of PTR2 in the mobilization of seed storage proteins during seed maturation.

PTR2 belongs to NRT1/PTR family (NPF) proteins. In addition to nitrate and di- and tri-peptides, some NPFs are involved in the uptake of other substrates, such as nitrite, chloride, glucosinolates, auxin, ABA, GA, and jasmonates (see review Corratge-Faillie and Lacombe 2017). Like PTR1 and PTR5 (Chiba et al. 2015), PTR2 might use different substrates, such as ABA and jasmonates. In the present study, we did not find any evidence that water uptake was influenced by these substrates, including IAA, GA, ABA, and glucose. This rules out the possibility that the transport activity of other substrates is involved in the observed phenotype, namely, water uptake driven seed germination. It is also unlikely that the post-germination process would be controlled by PTR2 transport activity, considering that the mutant phenotype was not observed during post-germination or afterwards during the growth and development stages.

*PTR2* transcripts (Rentsch et al. 1995; Dekkers et al. 2013; Supplementary Fig. S4) and 12S globulins (Pang et al. 1988) are stored in both the endosperm and embryo during seed maturation. Furthermore, a transient increase in *PTR2* expression during the transition from TR to ER is sensitive to cordycepin, a transcription inhibitor. Thus, PTR2 in germinating seeds is likely activated either post-transcriptionally or transcriptionally. While post-transcriptional activation of PTR2 could occur in both seed tissues, re-repression of ABI4-dependent transcriptional suppression of *PTR2* could occur only in the embryo (Fig. 5) since ABI4 is mostly active in the embryo, unlike ABI3 and ABI5, which function in both the embryo and endosperm during late phase II and early phase III. Accordingly, ABI4 seems to act as a spatiotemporal regulator of *PTR2* expression necessary for water uptake-dependent ER. The expression of *ABI4* is induced by high endogenous ABA levels during seed dormancy or exogenously supplied ABA, leading to the inhibition of ER. Thus, transient regulation of *ABI4* during the TR induction phase, when ABA level is low, implies the presence of an upstream regulator of *ABI4*. The DELLA protein RGL2 and the MYB protein MYB96 (Penfield et al. 2005, 2006; Lee et al. 2015; see review Gazzarrini and Tsai 2015) might act as putative regulators of *ABI4*, although this needs to be confirmed in a future study. Additionally, the redundant regulation of *PTR2* by ABI3 and ABI4 (Giraudat et al. 1992; Finkelstein et al. 1998; Finkelstein and Lynch 2000) should be also tested.

Taken together, our results suggest that disruption of PTR2 function results in lower water content in the endosperm and embryo during early seed germination in Arabidopsis. Tonoplast-localized PTR2 is likely activated post-transcriptionally upon water imbibition prior to TR and transcriptionally by ABI4 prior to onset of ER. In the expanding embryo, ABI4 may directly repress *PTR2* expression by binding to CCAC motifs in its promoter region. Further work will be required to demonstrate directly the osmotic potential build-up by PTR2-dependent increases in cytosolic di- and tri-peptides in germinating seeds.

## Electronic supplementary material

Below is the link to the electronic supplementary material.

Electronic supplementary material 1 (PPTX 17305 kb)

Electronic supplementary material 2 (DOCX 22 kb)

## Abbreviations

ABA: Abscisic acid
ABI: ABA insensitive
ER: Endosperm rupture
Glc: Glucose
PTR: Peptide transporter
TFs: Transcription factors
TR: Testa rupture
WT: Wild type
