# Supplementary material for: Peptide transporter2 (PTR2) enhances water uptake during early seed germination in Arabidopsis thaliana
Source: Plant Mol Biol. 2020 Jan 29;102(6):615–24. doi: 10.1007/s11103-020-00967-3 (PMC7062858; doi:10.1007/s11103-020-00967-3)
Supplement: Supplementary file 1 — Electronic supplementary material 1 (PPTX 17305 kb) [file 11103_2020_967_MOESM1_ESM.pptx]

## Slide 1
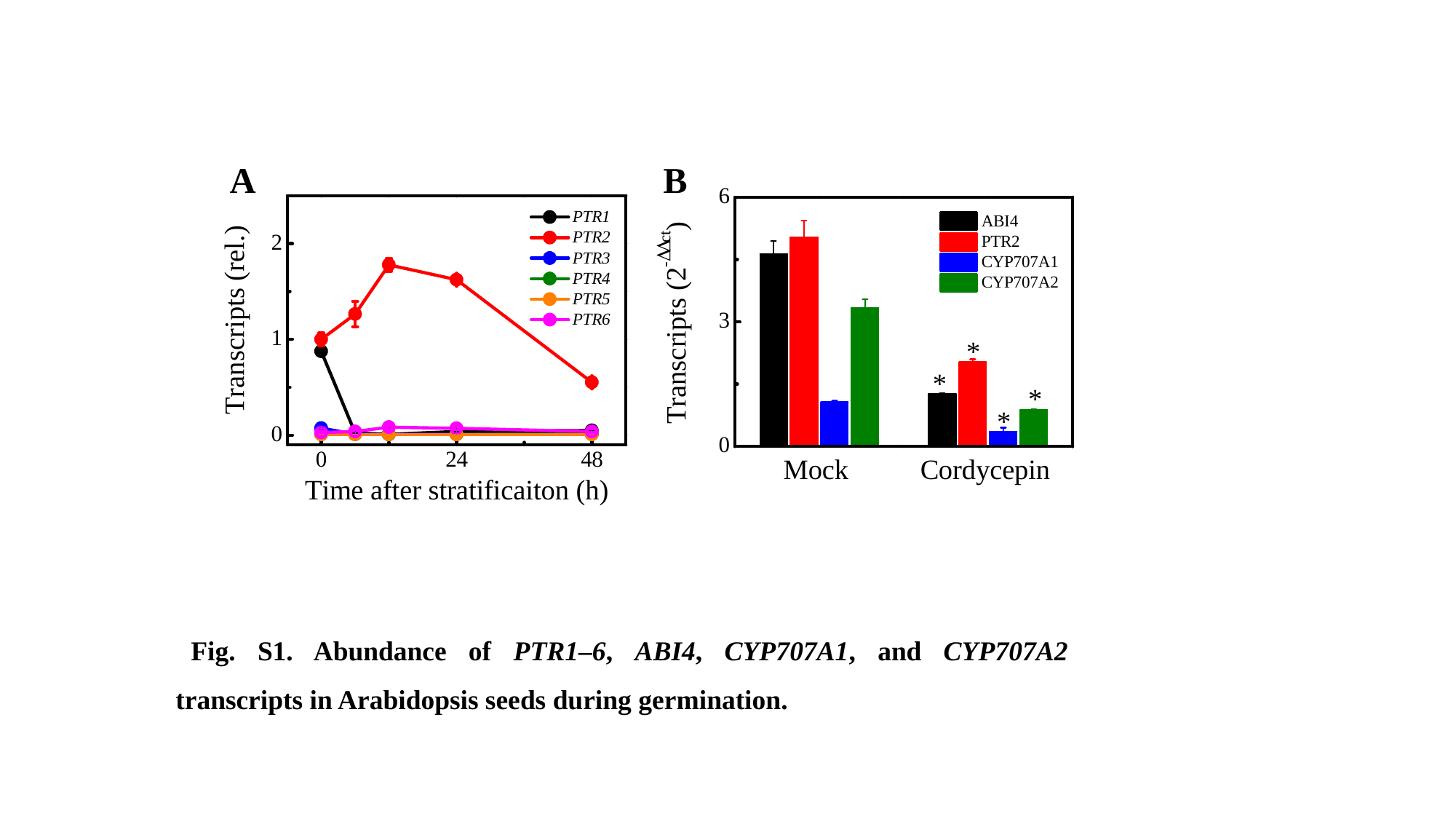

A B
Fig. S1. Abundance of PTR1–6, ABI4, CYP707A1, and CYP707A2 transcripts in Arabidopsis seeds during germination.

## Slide 2
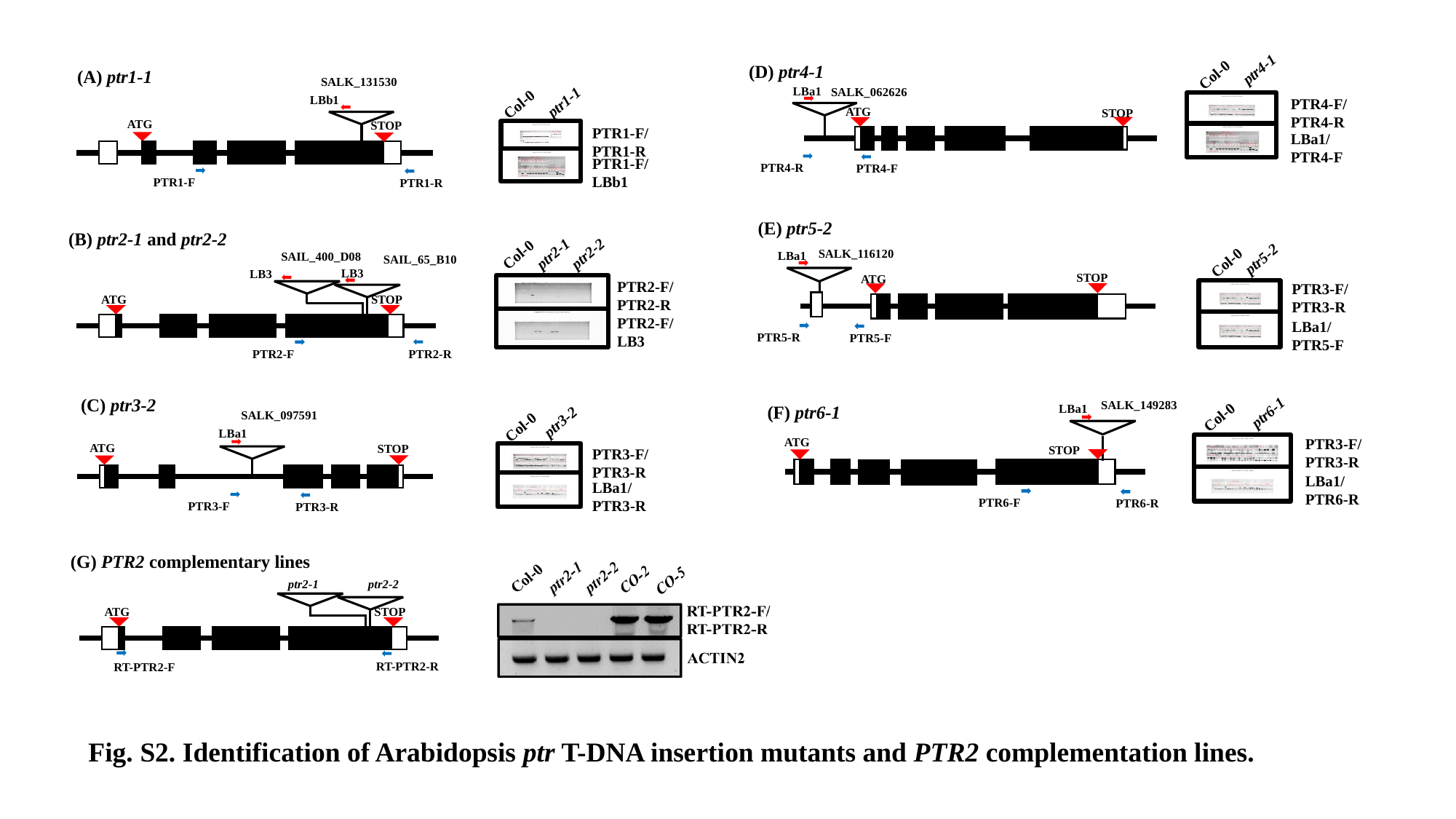

ptr4-1
Col-0
PTR4-F/
PTR4-R
LBa1/
PTR4-F
(D) ptr4-1
(A) ptr1-1
 SALK_131530
LBb1
ATG
STOP
PTR1-F
PTR1-R
LBa1
SALK_062626
ATG
STOP
PTR4-R
PTR4-F
ptr1-1
Col-0
PTR1-F/
PTR1-R
PTR1-F/
LBb1
(E) ptr5-2
(B) ptr2-1 and ptr2-2
ptr2-1
ptr2-2
Col-0
PTR2-F/
PTR2-R
PTR2-F/
LB3
SALK_116120
LBa1
STOP
ATG
PTR5-R
PTR5-F
SAIL_400_D08
 SAIL_65_B10
LB3
LB3
ATG
STOP
PTR2-F
PTR2-R
ptr5-2
Col-0
PTR3-F/
PTR3-R
LBa1/
PTR5-F
(C) ptr3-2
SALK_149283
LBa1
ATG
STOP
PTR6-F
PTR6-R
(F) ptr6-1
ptr6-1
Col-0
PTR3-F/
PTR3-R
LBa1/
PTR6-R
 SALK_097591
LBa1
ATG
STOP
PTR3-F
PTR3-R
ptr3-2
Col-0
PTR3-F/
PTR3-R
LBa1/
PTR3-R
(G) PTR2 complementary lines
ptr2-1 ptr2-2
ATG
STOP
RT-PTR2-F
RT-PTR2-R
Fig. S2. Identification of Arabidopsis ptr T-DNA insertion mutants and PTR2 complementation lines.

## Slide 3
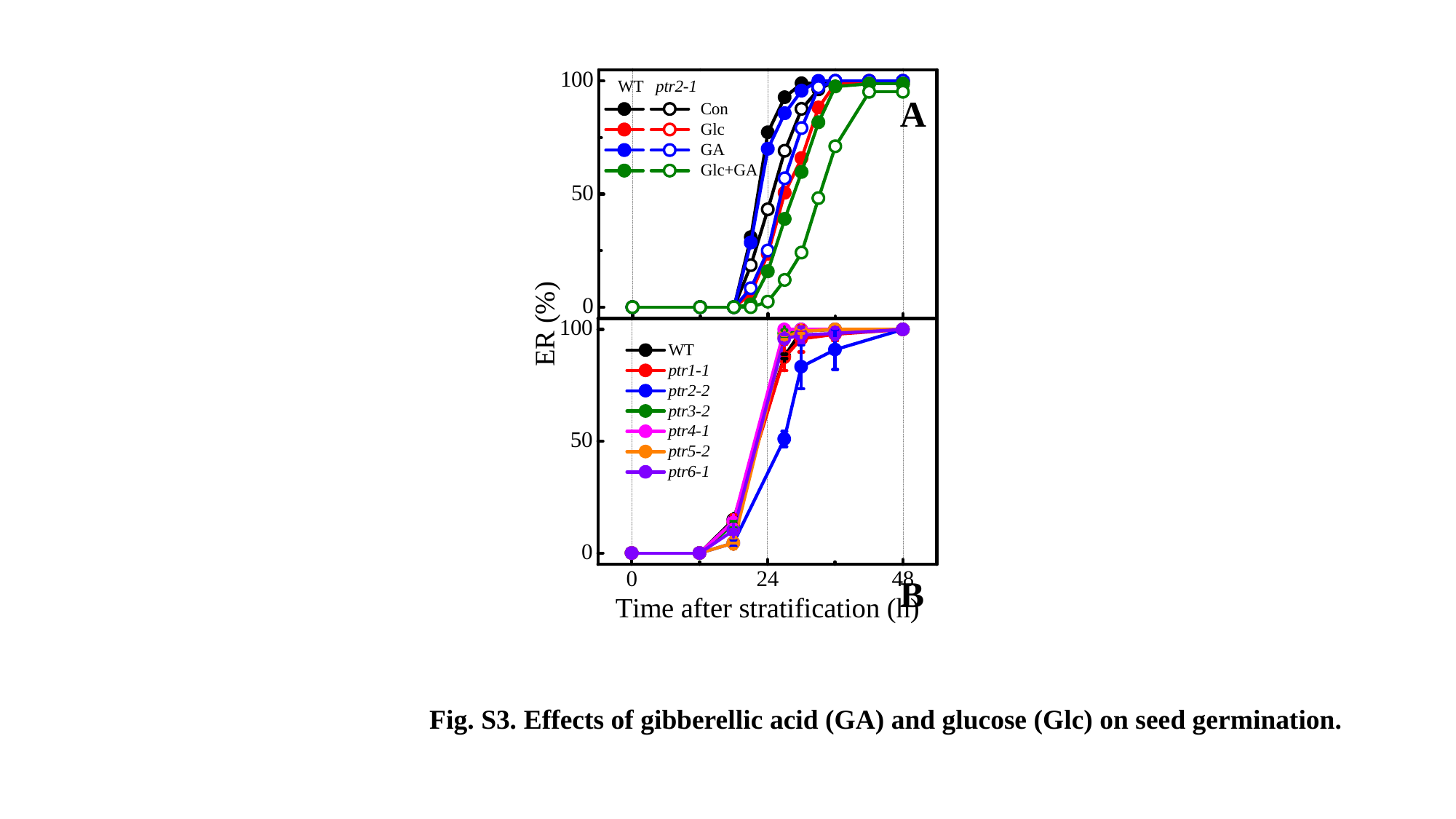

A
B
Fig. S3. Effects of gibberellic acid (GA) and glucose (Glc) on seed germination.

## Slide 4
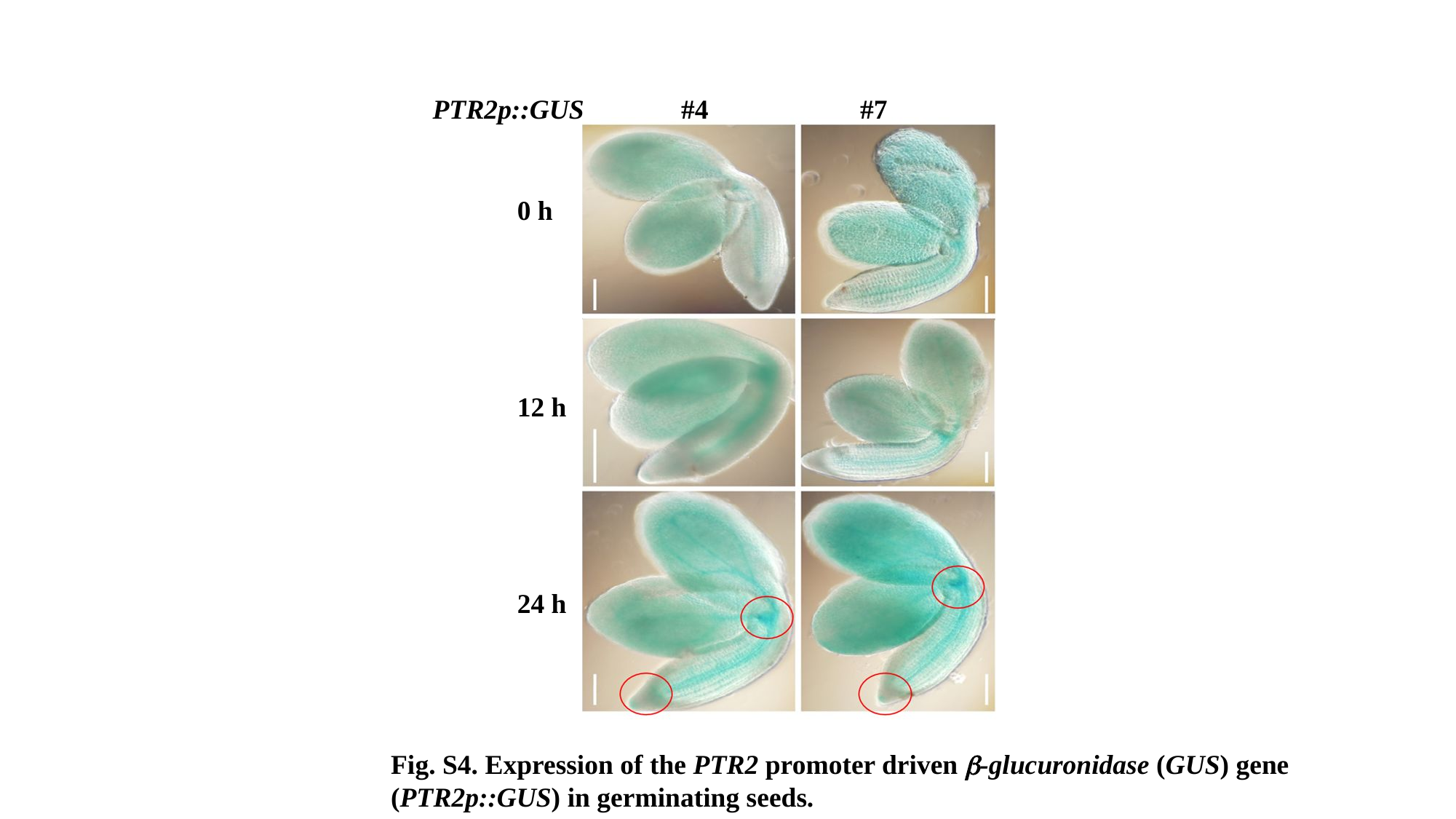

PTR2p::GUS #4 #7
0 h
12 h
24 h
Fig. S4. Expression of the PTR2 promoter driven b-glucuronidase (GUS) gene (PTR2p::GUS) in germinating seeds.

## Slide 5
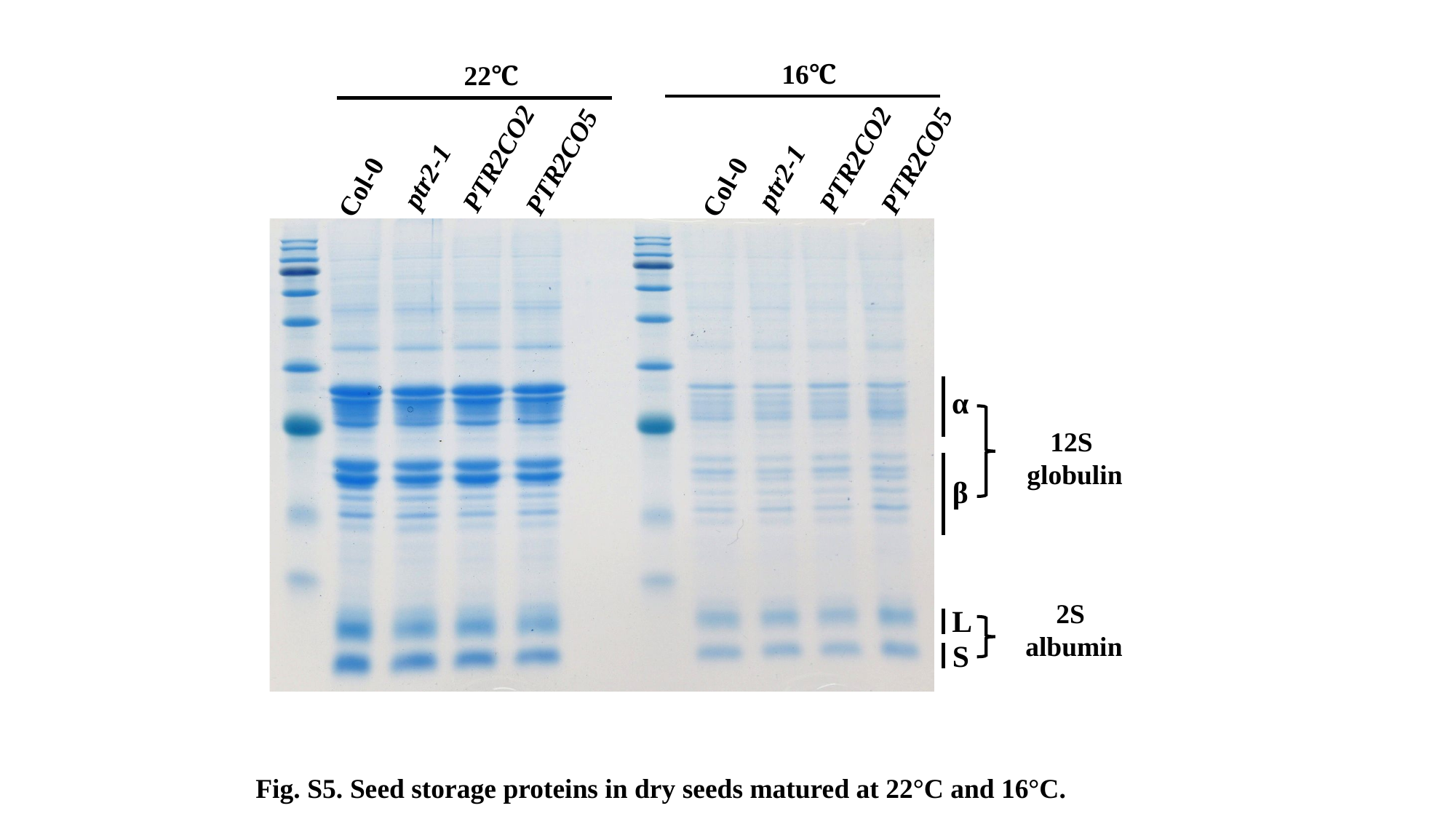

16℃
22℃
PTR2CO2
PTR2CO2
PTR2CO5
PTR2CO5
ptr2-1
ptr2-1
Col-0
Col-0
α
12S
globulin
β
2S
albumin
L
S
Fig. S5. Seed storage proteins in dry seeds matured at 22°C and 16°C.
