# Supplementary material for: Peptide transporter2 (PTR2) enhances water uptake during early seed germination in Arabidopsis thaliana
Source: Plant Mol Biol. 2020 Jan 29;102(6):615–24. doi: 10.1007/s11103-020-00967-3 (PMC7062858; doi:10.1007/s11103-020-00967-3)
Supplement: Supplementary file 2 — Electronic supplementary material 2 (DOCX 22 kb) [file 11103_2020_967_MOESM2_ESM.docx]

**SUPPLEMENTARY DATA**

**Table S1.** List of primers used in this study.

| **Primer name^a^** | **Primer sequence (5'→3')** |
| --- | --- |
| *Mutant isolation* | |
| PTR1_F | TGCTGTGAAAAGATTGGCCTAC |
| PTR1_R | GCAATGGCAAAATATAGGAGG |
| PTR2_F | CTTGACAAAGCCGCTGTTATC |
| PTR2_R | TCTTCACATTTCCATGGAAGG |
| PTR3_F | GAACAGTGTTGGCGAAGAGAG |
| PTR3_R | GAACCAACGACATGAAGGTTC |
| PTR5_F | CTTTCTTCATCAATGGAAGCC |
| PTR5_R | TCATTTAAGCGGTTCGAAAAC |
| PTR4_F | TTCTTTTGCGTGGTAGGTTTG |
| PTR4_R | TGTGACTTCACGAAGAAACAG |
| PTR6_F | TATCCGGATATCCGTTATCCG |
| PTR6_R | CAGCTGTAGAAGACAACTCGG |
| LBa1 | TGGTTCACGTAGTGGGCCATCG |
| LBb1 | TGGTTCACGTAGTGGGCCATCG |
| LB1 | GCCTTTTCAGAAATGGATAAATAGCCTTGCTTCC |
| *Transient Expression* | |
| PTR2-GUS_F | GTTCTGCAGAGGCTCTTGTGTGTA |
| PTR2-GUS_R | GTGAATTCAGAGGTCTTGCTTCTT |
| PTR2-GFP_F | CATCTAGAATGGGTTCCATCGAAG |
| PTR2-GFP_F | CATCTAGAATGGGTTCCATCGAAG |
| *qRT-PCR or RT-PCR* | |
| TR2-RT_F | ATGGGTTCCATCGAAGAA |
| TR2-RT_R | CGACGAAGCTTTCTTTTG |
| ACTIN2_F | ATTCAGATGCCCAGAAGTCTTGTT |
| ACTIN2_R | GAAACATTTTCTGTGAACGATTCC |
| PTR2_F | CCCCAGTTCCCATATCCGT |
| PTR2_R | CCTTCTTGCCCATTTCTTGTTGTA |
| ABI4_F | CAATAACTCATCCACCGCC |
| ABI4_R | ATCCCAAATACTCCCCCA |
| ABA1_F | CATTGTGATCCCTTCGTCTC |
| ABA1_R | TTCGGTGTTGCTCTATATCTTC |
| ABA2_F | TGTCAACGAACACTGAATCTTC |
| ABA2_R | TGGAACAGACGAACAATGC |
| ABA3_F | GGTTCTGCTTCATTCCTGAG |
| ABA3_R | CCCGTTTCCATGTCGTAAAG |
| ABA4_F | TTCAGTCCAATTCTTACACCG |
| ABA4_R | GTTCCAACCGCAAATACAC |
| CYP707A1_F | CCCCAACATCCTCTCTTT |
| CYP707A1_R | CCAAACTCCCACTCCCTC |
| CYP707A2_F | CGAATGGTGTTAGTGAGC |
| CYP707A2_R | GAGGAAAGGGTTTGGAGG |
| CYP707A3_F | CAAAGGGATGGAAAGTAC |
| CYP707A3_R | TTAGTGGTGAGATGATGG |
| CYP707A4_F | CTCCACCATTTAGTTTCC |
| CYP707A4_R | CTCTCCTCCCTTCACTTCCC |
| BG1_F | CAAGAGTGTGGATGGATAC |
| BG1_R | CAATGATAACTTCTGGGTC |
| BG2_F | GTTGGAAATGAGGTGAAA |
| BG2_R | GAAGTAAGGGTAGAGATT |
| UGT71B6_F | TGAGGAACAAGTGAGAGAA |
| UGT71B6_R | CGACGGAGAGACCAAAG |
| UGT71B7_F | ATACTCCTCCTGTTTATCC |
| UGT71B7_R | TCTCTCTTACTTGTTCCTC |
| UGT71B8_F | GTCTCTTCACTCTAGT |
| UGT71B8_R | GACCACTTCTCTCAAG |
| AP2M_F | CCAGATTCAACTCCGAGAAGA |
| AP2M_R | TAACCACGACCCCAAGG |
| *Electrophoretic mobility shift assay (EMSA)* | |
| PTR2-P1_F | GAGTCCCCAAACGATTTACTGA |
| PTR2-P1_R | AGAGAAGTAATTAACAAAACTC |
| PTR2-P2_F | TTGTGGAATCAATCAAAGTTGG |
| PTR2-P2_R | TCAGCAAAATTAGGAGAAATGG |
| PTR2-P3_F | CTCTTGGGTTGTGGAGAATATT |
| PTR2-P3_R | CCAACTTTGATTGATTCCACAA |
| PTR2-P4_F | CCATC CCATTCAAATTGTCCA |
| PTR2-P4_R | GAGTC AGATTATATAGGTTTG |
| ABI4-GST_F | ACTGTCGACCCATGGACCCTTTAGCTTCC |
| ABI4-GST_R | GCTCTCGAGAGGGGTTAAGTTGAGCTG |
| ABI5_F | CTCCGGCGGCTTTTAAACT |
| ABI5_R | CAGTCTTCTAATCCAAGATCTTG |

^a^“_F” and “_R” indicate forward and reverse primers, respectively.
